# Closing the antimicrobial stewardship gap - a call for LMICs to embrace the global antimicrobial stewardship accreditation scheme

**DOI:** 10.1186/s13756-024-01371-y

**Published:** 2024-02-14

**Authors:** Bashar Haruna Gulumbe, Mohammed Bashar Danlami, Abdulrakib Abdulrahim

**Affiliations:** Department of Microbiology, Faculty of Science, Federal University Birnin-Kebbi, Birnin-Kebbi, Kebbi State Nigeria

**Keywords:** Antimicrobial resistance, LMICs, Antimicrobial stewardship, ASP, AMR and Global Antimicrobial Stewardship Accreditation Scheme

## Abstract

The escalating challenge of antimicrobial resistance (AMR) poses a considerable concern for global health, particularly impacting low- and middle-income countries (LMICs). This article highlights the critical importance of tackling AMR in LMICs by adopting the Global Antimicrobial Stewardship Accreditation Scheme (GAMSAS). GAMSAS is portrayed as a holistic and sustainable strategy for antimicrobial stewardship, extending beyond accreditation to include educational programs, capacity enhancement, improved surveillance, and support for AMS policy research. While acknowledging the global uptake of the scheme, the article highlights its preliminary phase of adoption in LMICs, particularly in high-AMR burden regions like Sub-Saharan Africa. The piece stresses the imperative for LMICs to integrate GAMSAS, underscoring its significance in optimizing antimicrobial usage and patient health outcomes. It advocates for an all-encompassing approach that leverages international cooperation and sustained financial backing, crucial for the effective deployment and enduring success of antimicrobial stewardship efforts in these key areas.

The escalation of antimicrobial resistance (AMR) constitutes a critical concern for global health, particularly impacting low- and middle-income countries (LMICs), where limited resources and infrastructural challenges amplify the severity of this issue [1]. Within this setting, the Global Antimicrobial Stewardship Accreditation Scheme (GAMSAS) stands as a pivotal initiative, striving to enhance antimicrobial stewardship practices in a range of healthcare environments.

GAMSAS represents a model of sustainability and adaptability in antimicrobial stewardship (AMS), characterized by a points-based accreditation framework that relies on self-assessment surveys [2]. This innovative approach underpins the scheme’s effectiveness in advancing AMS practices. This innovative framework is designed to catalyze improvements in AMS practice. Central to its multifaceted objectives are identifying areas for enhancement, the augmentation of surveillance mechanisms for antimicrobial use and resistance, the elevation of awareness among healthcare stakeholders, and the bolstering of global AMS practice and policy research [2]. Integral to GAMSAS is the establishment of Centres of Excellence, which are instrumental in cultivating collaboration and disseminating exemplary AMS practices both regionally and nationally.

The roster of institutions that have achieved accreditation under GAMSAS is both diverse and prestigious. Notable among these are the Norton Healthcare Foundation in the United States and the Sheikh Khalifa Medical Centre SEHA in the United Arab Emirates, each recognized as a Level Three Centre of Excellence. Similarly, the Lagos University Teaching Hospital in Nigeria, accredited at Level Two, marks a significant milestone as the first institution in Africa to attain this status [2]. In Europe, St. Columcille’s Hospital in Ireland, the Imperial College Healthcare NHS Trust, The Walton Centre, Great Ormond Street Hospital for Children NHS Foundation Trust, University Hospitals of Coventry and Warwickshire, Cavan & Monaghan Hospital, and the Royal Papworth NHS Trust in the United Kingdom, have all been distinguished with varying levels of accreditation [2].

This commendable global participation notwithstanding, the engagement of LMICs in GAMSAS is still in its infancy. The pioneering achievement of Lagos University Teaching Hospital in Nigeria, which has emerged as the solitary beacon in Africa with a Level 2 accreditation, accentuates the necessity to expand the scheme’s reach, especially in areas heavily burdened by AMR, such as Sub-Saharan Africa [3]. This region, where AMR contributes to over a million deaths annually [3], urgently requires the heightened implementation of schemes like GAMSAS. Moreover, institutions like the American University of Beirut in Lebanon, the Instituto Nacional de Ciencias Medicas y Nutricion Salvador Zubiran in Mexico, and the Children Cancer Hospital Egypt 57,357 are actively working towards accreditation [2], showcasing the growing interest in robust antimicrobial stewardship across diverse global health economies. This expanding network of participation underscores the critical need for LMICs to embrace GAMSAS, thereby bridging the gap in global antimicrobial stewardship and fortifying the fight against AMR.

The challenges to implementing AMS in LMICs are multifaceted, including resource limitations, and infrastructural challenges, among others [4]. Despite these hurdles, the success stories of early adopters of GAMSAS demonstrate the potential for overcoming these obstacles with adequate support and commitment.

The strategic adoption of GAMSAS in LMICs offers a structured, evidence-based framework to improve antimicrobial use, enhancing patient outcomes and contributing to the reduction of AMR. The accreditation process itself is a tool for hospitals to benchmark against international standards, fostering a culture of continuous improvement and knowledge sharing [2]. Global collaboration and financial support are pivotal in facilitating GAMSAS adoption in LMICs. Contributions from entities like Baille Gifford and partnerships with international health bodies provide momentum [2]. Exchanging best practices and experiences from institutions that have successfully navigated the accreditation process offers invaluable guidance and motivation for others.

A successful stewardship program in LMICs, as envisioned by the GAMSAS, inherently encompasses more than just accreditation. It embodies a comprehensive strategy, integrating education, local capacity building, and continuous support [2]. This holistic approach is underpinned by collaborative efforts involving international health organizations, local governments, and healthcare providers [2]. Such partnerships are pivotal in ensuring the sustainability and efficacy of stewardship practices. GAMSAS’s multifaceted framework, thus, not only accredits but also actively fosters improvements in AMS practice, enhances surveillance, raises awareness, and supports practice and policy research in antimicrobial stewardship globally. Alongside these strategic endeavors, consistent and widespread financial support is another fundamental pillar. While philanthropic contributions have been instrumental in laying the groundwork, the scheme’s longevity and its capacity to foster ongoing improvements in AMS hinge on the establishment of more stable and enduring funding mechanisms. Such financial underpinnings are vital not only for achieving and maintaining accreditation but also for propelling AMS practices into a future that transcends the initial accreditation phase. This financial sustenance is key to expanding the reach and deepening the impact of GAMSAS, ensuring that the benefits of improved antimicrobial stewardship practices are felt broadly and sustainably, particularly in LMICs where the burden of antimicrobial resistance is most acute.

GAMSAS, therefore, presents a valuable framework for enhancing antimicrobial stewardship in LMICs. Yet, its effectiveness depends on a holistic, adaptable, and collaborative approach. The call for LMICs to embrace GAMSAS is a call for a comprehensive, sustainable, and collaborative effort to combat the AMR crisis in these crucial regions. As the world grapples with the growing threat of AMR, the success of initiatives like GAMSAS in LMICs will be instrumental in shaping a healthier, more resilient global community.

## Data Availability

Not applicable.
